# Clinical Randomized Comparison of Medetomidine and Xylazine for Isoflurane Balanced Anesthesia in Horses

**DOI:** 10.3389/fvets.2021.603695

**Published:** 2021-04-20

**Authors:** Alexandra Wiederkehr, Andrea Barbarossa, Simone K. Ringer, Fabiola B. Jörger, Marco Bryner, Regula Bettschart-Wolfensberger

**Affiliations:** ^1^Section Anesthesiology, Department of Clinical Diagnostics and Services, Vetsuisse Faculty, University of Zurich, Zurich, Switzerland; ^2^Department of Veterinary Medical Sciences, University of Bologna, Bologna, Italy; ^3^Clinic for Equine Surgery, Equine Department, Vetsuisse Faculty, University of Zurich, Zurich, Switzerland

**Keywords:** anesthesia, horse, isoflurane, medetomidine, alpha_2_-adrenergic agonists, equine, equisedative, recovery

## Abstract

**Introduction:** To assess drug plasma levels, preanesthetic sedation, cardiopulmonary effects during anesthesia and recovery in horses anesthetized with isoflurane combined with medetomidine or xylazine.

**Study design:** Prospective blinded randomized clinical study.

**Animals:** Sixty horses undergoing elective surgery.

**Methods:** Thirty minutes after administration of antibiotics, flunixine meglumine or phenylbutazone and acepromazine horses received medetomidine 7 μg kg^−1^ (group MED) or xylazine 1.1 mg kg^−1^ (group XYL) slowly intravenously (IV) and sedation was assessed 3 min later. Anesthesia was induced with ketamine/diazepam and maintained with isoflurane in oxygen/air and medetomidine 3.5 μg kg^−1^ h^−1^ or xylazine 0.69 mg kg^−1^ h^−1^. Ringer's acetate 10 mL kg^−1^ h^−1^ and dobutamine were administered to maintain normotension. All horses were mechanically ventilated to maintain end-tidal carbon dioxide pressures at 45 ± 5 mmHg (5.3–6.7 kPa). Heart rate (HR), invasive arterial blood pressures, inspired and expired gas compositions, pH, arterial blood gases, electrolytes, lactate and glucose were measured. For recovery all horses received intramuscular morphine 0.1 mg kg^−1^ and medetomidine 2 μg kg^−1^ or xylazine 0.3 mg kg^−1^ IV. Recovery was timed and scored using three different scoring systems. Plasma samples to measure medetomidine and xylazine concentrations were collected at predetermined timepoints. Repeatedly measured parameters were analyzed using a two-way repeated-measures analysis of variance for differences between groups and over time; *p* < 0.05 was considered statistically significant.

**Results:** Mean arterial blood pressures (MAP) stayed within normal ranges but were higher (*p* = 0.011) in group XYL despite significant lower dobutamine doses (*p* = 0.0003). Other measured parameters were within clinically acceptable ranges. Plasma levels were at steady state during anesthesia (MED 2.194 ± 0.073; XYL 708 ± 18.791 ng mL^−1^). During recovery lateral recumbency (MED 42.7 ± 2.51; XYL 34.3 ± 2.63 min; *p* = 0.027) and time to standing (MED 62.0 ± 2.86; XYL 48.8 ± 3.01 min; *p* = 0.002) were significantly shorter in group XYL compared to group MED. Recovery scores did not differ significantly between groups.

**Conclusion and Clinical Relevance:** In horses anesthetized with isoflurane and medetomidine or xylazine, xylazine maintained higher MAP, reduced the dobutamine consumption and recovery time, whilst overall recovery quality was unaffected.

## Introduction

Volatile anesthetic agents are commonly used in horses undergoing surgery; however, they cause cardio-respiratory depression (1) and they contribute to the equine anesthesia-associated morbidity and mortality (2, 3). Therefore, a balanced anesthetic protocol with partial intravenous anesthesia (PIVA) using one or more complementary agents is a common concept in modern equine general anesthesia (4–6). The aim of PIVA is to improve analgesia and to reduce the amount of the volatile anesthetic agents (7), in order to maintain good intraoperative cardiopulmonary function followed by smooth and coordinated recovery (4, 5, 8). Various alpha_2_-adrenergic agonists constant rate infusions (CRI) have been used in anesthetized horses for this purpose (9–13).

Half-lives of medetomidine (4.14 min) (14) and xylazine (5.9 min) (15) are relatively short and onset of action of the drugs is quick. Both drugs have been shown to result in constant plasma levels when administered as CRIs preceded by a bolus (15, 16).

Medetomidine, a potent, short acting, highly selective and specific alpha_2_-adrenergic agonist [selectivity ratio (alpha_2_:alpha_1_) 1,620:1] (17), has been shown to reduce the minimum alveolar concentration of isoflurane and provide potent analgesia (5, 18).

The reported side-effects after injection of a single IV bolus (dose) of medetomidine in non-anesthetized horses and ponies are brady-arrhythmias, increase in systemic vascular resistance and arterial blood pressure, and decrease of cardiac output (14, 19, 20). However, these effects are transitory and are not worsened by a following infusion (19). Even though it is not registered for horses in Europe or the United States, there are numerous reports about its use in equine clinical practice (6, 18, 21, 22). The administration of medetomidine CRI produced better recoveries than lidocaine or S-ketamine CRIs in isoflurane anesthetized horses (1, 23).

Xylazine, another alpha_2_-adrenergic agonist registered for horses also provides sedation, analgesia and muscle relaxation (24). It is the least selective alpha_2_-agonist [selectivity ratio (alpha_2_:alpha_1_) 160:1] and widely used as a premedicant (16, 25), but reports about its use as CRI in balanced anesthesia regimes are sparse (11).

Cardiopulmonary effects following xylazine bolus administration are also similar to other alpha_2_-adrenergic agonists, but during CRI the effects are minimal in particular if compared to romifidine CRI (26). In comparison to medetomidine cardiovascular effects of xylazine have been demonstrated in ponies to be less and shorter lasting (27). However, it remains to be tested if the dose rates investigated were equipotent. A bolus administration of IV xylazine at 0.5 and 1.0 mg kg^−1^ reduced isoflurane MAC by 25 and 34%, respectively (28).

The aim of the present study was to compare the intraoperative cardiovascular effects and recovery of xylazine vs. medetomidine isoflurane PIVA in horses undergoing elective surgery. The hypothesis of our study was that 7 μg kg^−1^ medetomidine followed by 3.5 μg kg^−1^ h^−1^ results in similar effects as 1.1 mg kg^−1^ xylazine followed by 0.69 mg kg^−1^ h^−1^.

## Materials and Methods

### Study Design and Animals

This prospective randomized blinded clinical study was performed with the ethical approval of the local committee for animal experimentation (ZH176/17). Written owner's consent was obtained.

Sixty client-owned horses of various breeds presented for elective surgeries were included in the study. This number was considered adequate to ascertain that the doses of medetomidine and xylazine tested were suitable regarding their influence on the concentration of isoflurane necessary to maintain anesthesia. In order to detect if isoflurane is increased from 1.1 ± 0.135 to 1.2% with a power of 0.8 and an alpha error of 0.05, 29 horses in each group are necessary. Inclusion criteria were body weight (≥200 kg), age (2–20 years), physical status American Society of Anesthesiologists 1 or 2. Exclusion criteria were surgery of the head, need of assisted recovery and general anesthesia in the previous 6 months. Food, but not water, was withheld 8–12 h prior to anesthesia. Each patient was randomly assigned to either group MED (medetomidine) or XYL (xylazine) at the beginning of the experiment, using one of sixty previously prepared opaque envelopes (containing group allocation), opened by a person who was not involved in the study. Demographic data was recorded on a separate data sheet (AW). All anesthetic procedures were performed by the same experienced anesthetist (AW), who was unaware of the treatment at any time until the end of the study.

### Study Protocol

Prior to sedation, the skin over one jugular vein was clipped and aseptically prepared. After infiltration with mepivacaine 2% (Mepivacain HCL 2%; Sintetica S.A., Switzerland), a 14 gauge catheter (Secalon-T with FlowSwitch; Argon Critical Care Systems, Singapore) was placed.

At 30–60 min before induction of anesthesia, patients in both groups received 30'000 I.E. kg^−1^ of penicilline Na^+^ (Penicillin Natrium Streuli ad us. vet.; Streuli Pharma AG, Switzerland), 6.6 mg kg^−1^ of gentamicine (Genta 10%.; CP-Pharma, Switzerland) intravenously (IV). Horses undergoing soft tissue surgeries received flunixin meglumine 1.1 mg kg^−1^ IV (Fluniximin ad us. vet.; Graeub AG, Switzerland) and horses undergoing orthopedic surgeries received phenylbutazone 4 mg kg^−1^ IV (Butadion ad us. vet.; Streuli Pharma AG, Switzerland). All horses received 0.03 mg kg^−1^ of acepromazine (Prequillan ad us vet.; Arovet AG, Switzerland) IM. Immediately before induction of anesthesia, horses were sedated with medetomidine 7 μg kg^−1^ IV (Medetor® ad us. vet.; Virbac AG, Switzerland) or xylazine 1.1 mg kg^−1^ IV (Xylazin 2%; Streuli Pharma AG, Switzerland) depending on group allocation. Medication were prepared by a veterinarian not involved in the study and diluted with sodium chloride to a total volume of 40 mL. One-third of the dose was administered as a bolus in the stable, then the mouth was rinsed and the horse walked to the induction area, where the remaining two-thirds of sedative were injected IV slowly over 2 min. Three minutes after the end of injection, depth of sedation was assessed according to criteria published by Taylor et al. (29): where sedation is indicated by head hight lower than withers (eyes as benchmark), atonic lower lip, no reaction to snapping fingers. If one or more than one condition indicating sedation was not present, a supplemental dose of medetomidine 1 μg kg^−1^ or xylazine 0.16 mg kg^−1^ was administered IV, depending on group allocation. Sedation was re-evaluated after 3 min. This procedure was repeated if necessary. The total amount of time required for sedation was recorded.

Anesthesia was induced with IV diazepam 0.02 mg kg^−1^ IV (Valium® 5 mg; Roche Pharma AG, Switzerland) and ketamine 2.2 mg kg^−1^ (Ketanarkon 100 ad us. vet.; Streuli Pharma AG, Switzerland) mixed in one syringe. This was considered as anesthesia time point zero (*t*_0_). Following induction of anesthesia and tracheal intubation (silicone tubes, internal diameter 18–30 mm), the horses were hoisted onto a padded surgery table and the endotracheal tube attached to a large animal circle system (Tafonius; Hallowell Engineering & Manufacturing Corp., MA, USA). Isoflurane was delivered using a precision vaporizer (Tec 7, Rothacher Medical GmbH, Heitenried, Switzerland). The carrier gas for isoflurane (IsoFlo®, Provet AG, Switzerland) delivery consisted of a mixture of oxygen and air with an initial inspiratory fraction of oxygen (F_I_O_2_) of 0.45–0.55. Every 5 min, the depth of anesthesia was assessed and isoflurane supply readjusted based on judgement by the main investigator, who was unaware of the treatment. Isoflurane was delivered to the end-tidal concentration (F_E_'Iso) required to keep the patient immobile, to prevent nystagmus and hypertension, to provide muscle relaxation and to allow evocation of sluggish palpebral reflexes. Mechanical ventilation was commenced (tidal volume 10–12 mL kg^−1^, I:E ratio 1:2.5–3, peak airway pressure below 25 cmH_2_O, variable respiratory rate) to an expired partial pressure of CO_2_ between 45 ± 5 mmHg (5.3–6.6 kPa). Artificial ventilation was started immediately after connection to the breathing system and continued until the end of anesthesia. A urinary catheter was placed in all horses immediately following anesthesia induction and was left in place until the horse had recovered from anesthesia. The amount of urine produced was measured beginning as soon as the urinary catheter was placed until the disconnection of the patient from the large animal anesthesia machine. The urinary production during recovery was not measured. Medetomidine 3.5 μg kg^−1^ h^−1^ or xylazine 0.69 mg kg^−1^ h^−1^ for group MED and group XYL, respectively, were started immediately after positioning the animal on the surgery table, delivered IV by an infusion pump (Phoenix D; Schoch Electronics, Switzerland). A 20 gauge catheter (SURFLO®, Terumo Europe N.V., Belgium) was placed percutaneously in a transverse facial artery for the measurement of direct arterial blood pressures and to collect blood for arterial blood gas analysis and plasma concentrations of medetomidine and xylazine. A non-distensible extension line filled with heparinized saline solution was connected to the arterial catheter and to an electronic transducer (Monitoring Kit Transpac IT; Abbott Ireland, Ireland) placed at level of shoulder joint (dorsal recumbence) or manubrium sternum (lateral recumbence), zeroed to atmospheric pressure and arterial blood pressures were obtained. HR, respiratory rate (RR), end expiratory carbondioxide tension (P_E_'CO_2_), expiratory isoflurane concentration (E_T_Iso) as well as arterial blood pressures were monitored continuously with a multiparameter monitor (Datex-Ohmeda-Cardiocap/5') and manually recorded every 5 min. After anesthesia induction, dobutamine (Dobutrex; Teva Pharma AG, Switzerland) was started at a rate of 40 μg kg^−1^ h^−1^ via an infusion pump (Volumed® μVP5005; Arcomed, Switzerland). In order to keep the MAP values stable, the rate of dobutamine was readjusted every 5 min based on a prepared rate scale in dependence on the MAP (<65 mmHg: 70 μg kg^−1^ h^−1^; 65–70 mmHg: 60 μg kg^−1^ h^−1^; 71–75 mmHg: 50 μg kg^−1^ h^−1^; 76–80 mmHg: 40 μg kg^−1^ h^−1^; 81–85 mmHg: 30 μg kg^−1^ h^−1^; 86–90 mmHg: 15 μg kg^−1^ h^−1^; >90 mmHg: 0 μg kg^−1^ h^−1^). Intravenous crystalloids (Ringer-Laktat; Bichsel AG, Switzerland) were administered IV at a rate of 8–11 mL kg^−1^ h^−1^. If despite 70 μg kg^−1^ h^−1^ dobutamine the MAP remained below 65 mmHg or if the HR increased to a value higher than 1.5 times HR at *t*_30_, a bolus of 5 mL kg^−1^ lactated Ringer solution was administered over 10 min. This bolus application was repeated once. If MAP did not rise above 65 mmHg a 2 mL kg^−1^ IV bolus of tetrastarch (Voluven balanced 6%; Fresenius Kabi AG) was administered in addition to the dobutamine infusion. The tetrastarch bolus application was repeated until MAP > 65 mmHg. If nystagmus or incessant fighting against the ventilator occurred, ketamine 0.1 mg kg^−1^ IV was administered. In case of movement, thiopental 0.5 mg kg^−1^ IV (Thiopentalum natricum; Spedalia AG, Switzerland) was given every minute until movement stopped. At time points *t*_30_, *t*_60_, *t*_90_, *t*_150_, and *t*_210_, arterial blood samples were drawn anerobically using pre-heparinized syringes (BD A-Line; Becton, Dickinson and Co., UK), and analyzed immediately (RAPIDPoint®500; Siemens, Germany). Analysis included pH, arterial partial pressure of oxygen (PaO_2_) and carbon dioxide (PaCO_2_), base excess (BE) and electrolytes (Na^+^, K^+^, Ca^2+^, Cl^−^) as well as arterial lactate (Lac). If a PaO_2_ <80 mmHg (10.7 kPa) was detected, FIO_2_ was increased to 1.0. If a PaO_2_ <60 mmHg (8 kPa) was detected, salbutamol (Ventolin® GlaxoSmithKline AG, Müchenbuchsee, Switzerland) 2 μg kg^−1^ was administered intratracheally. Thirty minutes prior to the end of anesthesia, morphine 0.1 mg kg^−1^ (Morphine HCL 10 mg; Sintetica SA, Switzerland) was administered IM. At the end of surgery, all infusions and mechanical ventilation were stopped and horses were hoisted from the table to a padded recovery box. From the moment of disconnection of the horse from the anesthetic machine, the recovery timing was started. Ventilation was assisted using a demand valve (JD Medical, Phoenix, Arizona, USA) twice per minute until the horse started to breath spontaneously. Subsequently, medetomidine 2 μg kg^−1^ (group MED) or xylazine 0.3 mg kg^−1^ (group XYL) was administered IV slowly over 2 min. If at that timepoint horses either already moved or showed excessive nystagmus the post anesthesia dose of either medetomidine or xylazine was doubled. Oxygen was administered (15 L min^−1^) through the endotracheal tube (ETT). The horse's tracheas were extubated 15 min after disconnection of the horse from the anesthetic machine. Before removing the ETT, 5 mL phenylephrine 0.15% drops (Phenylephrin hydrochloridum 1.5 mg mL^−1^; Volksapotheke Schaffhausen, Switzerland) were instilled into each nasal cavity. Horses were allowed to recover without assistance. Times to attain sternal and standing positions were noted. The main anesthetist assessed recovery quality. Three different scoring systems (6, 30, 31) were used for assessment.

Plasma medetomidine and xylazine concentrations were determined from blood samples taken from the jugular vein not used for drug injection or (during anesthesia) from the arterial catheter. Blood was sampled before premedication, after sedation, *t*_30_, *t*_60_, *t*_90_, *t*_150_, *t*_210_, just before the postoperative sedation and 5–10 min after reaching standing position. Before each sampling, 10 mL of blood was withdrawn, then 6 mL of blood was collected into lithium-heparin containing vacutainers (6 mL Vacutainer®, Becton Dickinson, Meylan Cedex, France). This blood was centrifuged without delay and the plasma frozen at −80°C until analyses. Plasma samples were analyzed by liquid chromatography-tandem mass spectrometry (LC-MS/MS). For xylazine determination, a 200 μL aliquot of plasma was added with the internal standard (xylazine-d6) and 800 μL of acetonitrile, then 100 μL of the supernatant were transferred into a vial containing 900 μL of 0.1% formic acid aqueous solution prior to injection. As for medetomidine, internal standard medetomidine-d3 was added to 300 μL of plasma and a liquid-liquid extraction with 900 μL of ethyl acetate was performed. The dry residue of the supernatant was reconstituted in a 0.1% formic acid in water: acetonitrile 90:10 (*v/v*) solution and injected. The LC system was a Waters Acquity UPLC binary pump, equipped with a Waters BEH C18 (2.1 × 50 mm, 1.7 um) column (Waters, Milford, MA, USA). The mobile phase was a mixture of acetonitrile and 0.1% formic acid in water, at a flow rate of 0.3 mL min^−1^ under programmed conditions. The detector was a Waters Quattro Premier XE triple quadrupole mass spectrometer (Waters, Milford, MA, USA) with positive electrospray ionization source (ESI+), operating in multiple reaction monitoring (MRM) mode. The specific transitions observed for each analyte were: 221.0 → 89.8 and 163.7 for xylazine; 227.0 → 169.8 for xylazine-d6; 201.0 → 94.7 and 67.7 for medetomidine; 204.0 → 97.6 for medetomidine-d3. The two methods were validated in accordance with EMEA/CHMP/EWP/192217/2009 guidelines before the experiment.

### Statistical Analysis

Data distribution was tested with the Shapiro-Wilk test of normality. Depending on distribution, *t*-test or Mann-Whitney test was used to assess differences between groups in terms of weight, age, anesthesia duration, recovery times, intra-anesthetically administered lactated Ringer solution, dobutamine, injected ketamine or thiopental and scores. Repeatedly measured parameters were analyzed using a two-way repeated-measures analysis of variance (ANOVA) followed by Holm-Sidak multiple comparisons test to analyse differences between groups and over time. Chi-square test was used to evaluate significant group differences between required doses of sedation. HR and MAP analyses was restricted to time point *t*_20_-*t*_120_ and *t*_30_-*t*_120_, respectively. Significance was considered when *p* ≤ 0.05. Statistical analysis was performed using SigmaStat 3.5 (Systat Software, CA, USA). Results of parametric data are expressed as mean ± standard error of the mean and results of non-parametric data as median (range).

## Results

Sixty horses were randomly allocated into two groups (MED or XYL). Due to a mistake in the arrangement 29 horses were in group XYL and 31 horses in group MED.

**Figure 1:**
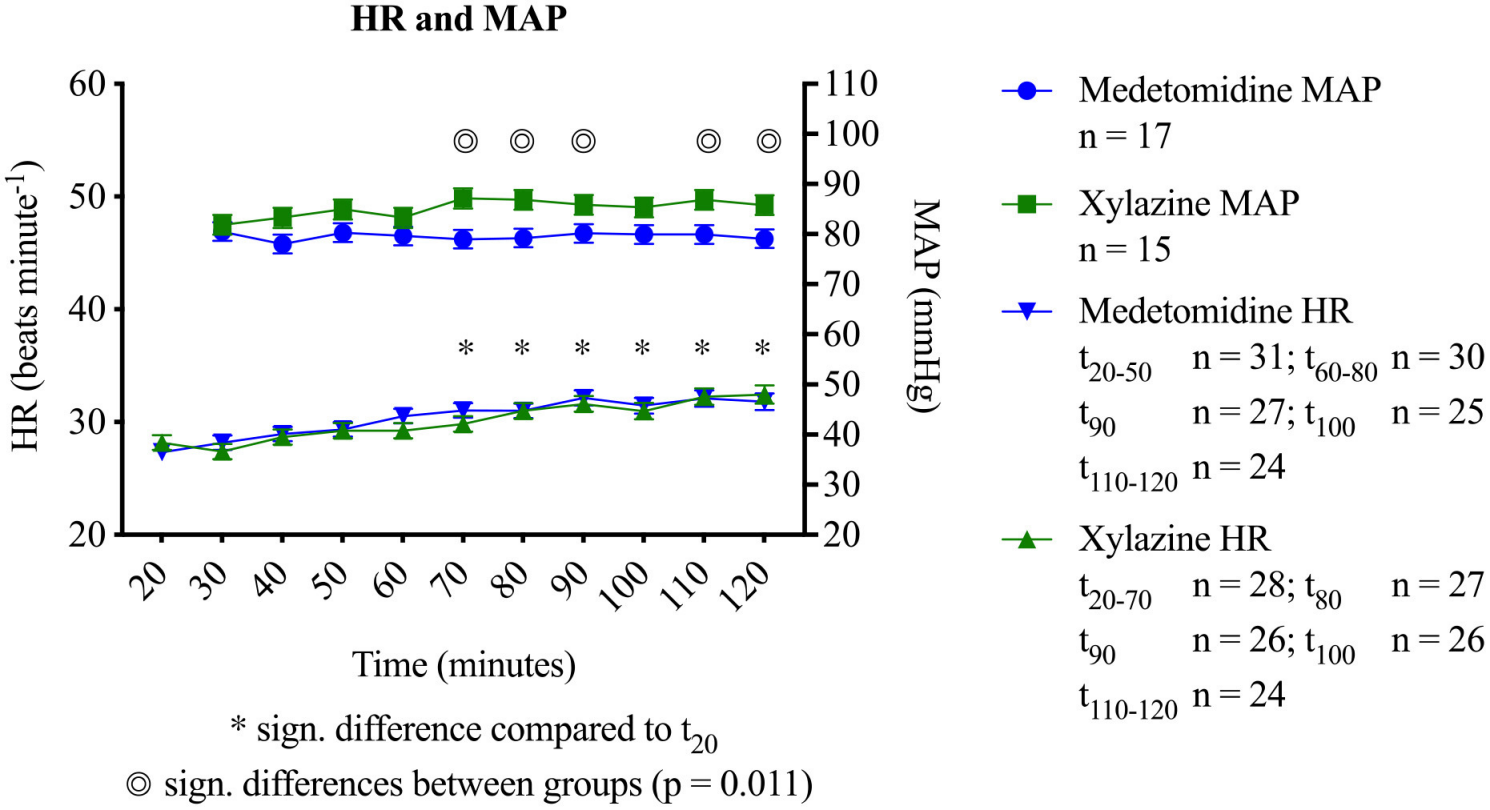
Heart rates (HR) and mean arterial blood pressures (MAP) in horses during medetomidine isoflurane (group MED) or xylazine isoflurane (group XYL) balanced anesthesia (mean ± SEM). Significant differences between groups is marked with a double circle (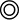). Time points with significant difference to *t*_20_ are marked with asterisk (*). Represented on the x-axis are minutes after anesthesia induction.

One horse in group XYL showed seizures of unknown origin during anesthesia and was completely excluded from data analyses. One horse in group MED needed treatment with salbutamol 2 μg kg^−1^ intratracheally but was excluded completely from data analysis due to technical problems 10 min later. Furthermore, one horse from each group had to be excluded from the recovery scoring due to intraoperative decisions for assisted recovery (XYL) or bleeding during recovery (MED). Otherwise, numbers of horses from which data originated are indicated in the corresponding table or figure (Tables 1–3 and Figure 1).

**Table 1:** Mean ± SEM of venous blood-gas values, pH, lactate concentrations, total hemoglobin, base excess in horses with medetomidine isoflurane (group MED) or xylazine isoflurane (group XYL) balanced anesthesia.

**Parameter**	**Group**	**Time point**			
		**B**	**I**	**R**	**S**
	n MED	31	31	29	29
	n XYL	28	27	28	25
P_v_CO_2_, mmHg (kPa)	MED	46.89 ± 0.73 (6.3 ± 0.1)	48.20 ± 0.73 (6.4 ± 0.1)	62.27 ± 0.76 (8.3 ± 0.1)^#^	52.11 ± 0.76 (6.9 ± 0.1)^#^
	XYL	46.58 ± 0.77 (6.2 ± 0.1)	49.14 ± 0.78 (6.6 ± 0.1)^#^	63.02 ± 0.77 (8.4 ± 0.1)^#^	53.54 ± 0.82 (7.1 ± 0.1)^#^
P_v_O_2_, mmHg (kPa)	MED	34.35 ± 0.80 (4.6 ± 0.1)	28.44 ± 0.81 (3.8 ± 0.1)^#^	47.62 ± 0.83 (6.3 ± 0.1)^*^^#^	33.64 ± 0.83 (4.5 ± 0.1)
	XYL	34.90 ± 0.83 (4.7 ± 0.1)	28.33 ± 0.85 (3.8 ± 0.1)^#^	44.90 ± 0.83 (6.0 ± 0.1)^*^^#^	33.85 ± 0.90 (4.5 ± 0.1)
pH	MED	7.41 ± 0.004	7.39 ± 0.004^#^	7.32 ± 0.004^#^	7.40 ± 0.004^#^
	XYL	7.41 ± 0.004	7.38 ± 0.004^#^	7.32 ± 0.004^#^	7.39 ± 0.004^#^
Lactate (mmol L^−1^)	MED	0.63 ± 0.16	0.69 ± 0.16	2.02 ± 0.17^#^	2.03 ± 0.17^#^
	XYL	0.62 ± 0.17	0.70 ± 0.17	1.58 ± 0.17^#^	1.80 ± 0.19^#^
Hb (g dL^−1^)	MED	12.61 ± 0.21	12.11 ± 0.20	10.99 ± 0.22^#^	11.60 ± 0.22^#^
	XYL	12.61 ± 0.22	11.69 ± 0.22^#^	10.68 ± 0.22^#^	12.06 ± 0.23
BE (mmol L^−1^)	MED	4.00 ± 0.29	2.64 ± 0.29^#^	4.01 ± 0.30	5.72 ± 0.30^#^
	XYL	3.41 ± 0.30	2.02 ± 0.31^#^	4.43 ± 0.30^#^	5.90 ± 0.32^#^

Significant differences (p < 0.05) between groups are indicated by

* *and significant changes compared to B in the same group are indicated by #*.

*P_v_O_2_, P_v_CO_2_, venous oxygen and carbon dioxide partial pressures; Lac, lactate concentration; Hb, total hemoglobin; BE, base excess; B, Baseline; I, Induction; R, Recovery; S, Standing*.

No significant differences in age (MED: 11.5 ± 0.8; XYL: 11.1 ± 1.0 years; *p* = 0.760); body weight (MED: 532 ± 14; XYL: 520 ± 19 kg; *p* = 0.599), breed (46 warmbloods; 5 thoroughbreds; 8 ponies), BCS (4 horses BCS 2/5; 47 horses BCS 3/5; 7 horses BCS 4/5; 1 horse BCS 5/5), sex (13 mares; 17 stallions; 29 geldings), type of recumbency (30 dorsal recumbency; 29 lateral recumbency), type of procedure (28 orthopedic procedures; 31 soft tissue procedure) or total duration of anesthesia (MED: 145.4 ± 6.3; XYL: 148 ± 6.8 min; *p* = 0.777) were identified between groups. During preoperative sedation six horses in group XYL showed tumbling before the full dose of xylazine was administered. Further administration of xylazine was stopped immediately and sedation criteria evaluated after 3 min. All six horses pervaded the criteria and anesthesia was induced. In both groups some horses (MED *n* = 4; XYL *n* = 2) did not fulfill the sedation criteria and required one or more supplemental dose. The median dose of sedation necessary to fulfill sedation criteria prior to anesthesia induction was 7 (7–10) μg kg^−1^ in group MED and 1.1 (0.8–1.3) mg kg^−1^ in group XYL. There was no significant difference in the time from the start of sedation to induction of anesthesia between groups [MED: 6 (3–8); XYL: 7 (4–14) min; *p* = 0.101].

No significant difference was found between groups regarding mean E_T_'Iso concentrations (MED: 1.1 ± 0.02; XYL: 1.1 ± 0.02%; *p* = 0.267). Nevertheless, group-independent changes over time were significant with E_T_'ISO increasing over time compared to *t*_20_.

Figure 1 displays MAP and HR. MAP was within the targeted values but significantly higher with xylazine compared to medetomidine at time points *t*_70_ (*p* = 0.007), *t*_80_ (*p* = 0.005), *t*_90_ (*p* = 0.017), *t*_110_ (*p* = 0.013) and *t*_120_ (*p* = 0.015). No differences between groups were detected for HR (*p* = 0.85). However, an increase in HR over time was observed for both groups (*p* < 0.001). Dobutamine requirement was significantly higher with medetomidine (MED: 36 ± 1.8; XYL: 24 ± 1.8 μg kg^−1^ h^−1^; *p* = 0.0003). Five horses in group MED required between one and three lactated Ringer solution boluses and one of them needed two boluses of tetrastarch, while in group XYL only three horses received one bolus of lactated Ringer solution to achieve the targeted values of MAP and HR. Results of venous and arterial pH and arterial blood gases are found in Tables 1, 2 and those of electrolytes, lactate and hemoglobin in Table 3. There were some changes over time and very minimal differences in arterial partial pressure of CO_2_ (P_a_CO_2_), venous partial pressure of O_2_ (P_v_O_2_), lactate, hemoglobin concentrations and Ca^2+^ between the groups (Tables 1–3).

**Table 2:** Mean ± SEM of arterial blood-gas values, pH, lactate concentrations, total hemoglobin, base excess in horses with medetomidine isoflurane (group MED) or xylazine isoflurane (group XYL) balanced anesthesia.

**Parameter**	**Group**	**Time point**			
		***t*_**30**_**	***t*_**60**_**	***t*_**90**_**	***t*_**150**_**
	n MED	31	30	26	8
	n XYL	27	27	26	10
P_a_CO_2_, mmHg (kPa)	MED	50.57 ± 0.49 (6.7 ± 0.07)	52.89 ± 0.51 (7.1 ± 0.07)^*^^#^	54.82 ± 0.56 (7.3 ± 0.07)^*^^#^	55.56 ± 1.10 (7.4 ± 0.15)^#^
	XYL	51.19 ± 0.52 (6.8 ± 0.07)	51.03 ± 0.54 (6.8 ± 0.07)^*^	52.11 ± 0.55 (6.9 ± 0.07)^*^	52.45 ± 0.96 (7.0 ± 0.13)
P_a_O_2_, mmHg (kPa)	MED	153.97 ± 5.16 (20.5 ± 0.69)	151.34 ± 5.45 (20.2 ± 0.73)	146.47 ± 5.90 (19.5 ± 0.79)	152.00 ± 11.40 (20.3 ± 1.52)
	XYL	151.03 ± 5.53 (20.1 ± 0.74)	148.56 ± 5.53 (19.8 ± 0.79)	155.82 ± 5.68 (20.8 ± 0.76)	145.30 ± 10.00 (19.4 ± 1.33)
pH	MED	7.35 ± 0.003	7.35 ± 0.003	7.35 ± 0.003	7.36 ± 0.006
	XYL	7.35 ± 0.003	7.36 ± 0.003	7.36 ± 0.003	7.38 ±0.006^#^
Lactate (mmol L^−1^)	MED	1.61 ± 0.03	1.83 ± 0.03^#^	1.9 ± 0.03^*^^#^	2.00 ± 0.06^#^
	XYL	1.47 ± 0.03	1.60 ± 0.03^#^	1.65 ± 0.03^*^^#^	1.56 ± 0.06^#^
Hb (g dL^−1^)	MED	11.32 ± 0.12	10.6 ± 0.12^#^	10.40 ± 0.13^#^	10.88 ± 0.25
	XYL	11.62 ± 0.12	10.76 ± 0.13^#^	10.35 ± 0.13^#^	9.52 ± 0.24^#^
BE (mmol L^−1^)	MED	2.37 ± 0.20	2.00 ± 0.21^#^	2.87 ± 0.23^#^	4.55 ± 0.44^#^
	XYL	1.56 ± 0.21	2.10 ± 0.22	2.50 ± 0.22^#^	4.05 ± 0.39^#^

Significant differences (p < 0.05) between groups are indicated by

* *and significant changes compared to t_30_ in the same group are indicated by #*.

*P_a_O_2_, P_a_CO_2_, arterial oxygen and carbon dioxide partial pressures; Lac, lactate concentration; Hb, total hemoglobin; BE, base excess; t_30−150_, timepoints in minutes after induction*.

**Table 3:** Mean ± SEM of electrolytes in horses with medetomidine isoflurane (group MED) or xylazine isoflurane (group XYL) balanced anesthesia.

**Parameter**	**Group**	**Time point**							
		**B (venous)**	**I (venous)**	***t*_**30**_ (arterial)**	***t*_**60**_ (arterial)**	***t*_**90**_ (arterial)**	***t*_**150**_ (arterial)**	**R (venous)**	**S (venous)**
	n MED	31	31	31	30	26	8	29	29
	n XYL	28	27	27	27	26	10	28	25
Na^+^ (mmol L^−1^)	MED	135.99 ± 0.31	135.94 ± 0.30					135.09 ± 0.31	135.83 ± 0.31
	XYL	135.81 ± 0.32	136.89 ± 0.32					134.99 ± 0.31^#^	136.77 ± 0.34
K^+^ (mmol L^−1^)	MED	3.58 ± 0.04	3.49 ± 0.04					3.71 ± 0.04^#^	3.91 ± 0.04^#^
	XYL	3.67 ± 0.04	3.55 ± 0.04					3.68 ± 0.04	3.98 ± 0.04^#^
Ca^2+^ (mmol L^−1^)	MED^*^	1.46 ± 0.007	1.45 ± 0.007					1.30 ± 0.007^*^^#^	1.28 ± 0.007^*^^#^
	XYL^*^	1.49 ± 0.007	1.48 ± 0.007					1.34 ± 0.007^*^^#^	1.31 ± 0.007^*^^#^
Cl^−1^ (mmol L^−1^)	MED	100.45 ± 0.24	100.58 ± 0.24					99.31 ± 0.25^#^	98.24 ± 0.25^#^
	XYL	100.39 ± 0.25	100.44 ± 0.26					99.18 ± 0.25^#^	98.28 ± 0.27^#^
Na^+^ (mmol L^−1^)	MED			134 ± 0.25	134.04 ± 0.26	134.51 ± 0.30^#^	134.54 ± 0.56^#^		
	XYL			133.26 ± 0.27	133.64 ± 0.28	134.32 ± 0.29	134.15 ± 0.50		
K^+^ (mmol L^−1^)	MED			3.6 ± 0.02	3.5 ± 0.02	3.50 ± 0.03	3.69 ± 0.05		
	XYL			3.54 ± 0.02	3.53 ± 0.03	3.53 ± 0.03	3.52 ± 0.05		
Ca^2+^ (mmol L^−1^)	MED			1.38 ± 0.005	1.34 ± 0.005^#^	1.32 ± 0.006^#^	1.29 ± 0.011^#^		
	XYL			1.4 ± 0.005	1.36 ± 0.006^#^	1.34 ± 0.006^#^	1.32 ± 0.010^#^		
Cl^−1^ (mmol L^−1^)	MED			99.77 ± 0.20	100.10 ± 0.20	100.12 ± 0.22	100.13 ± 0.43		
	XYL			99.61 ± 0.20	100.22 ± 0.21^#^	100.19 ± 0.22	101.00 ± 0.38^#^		

Significant differences (p < 0.05) between groups are indicated by

* *and significant changes compared to B or t_30_ in the same group are indicated by #*.

*Na^+^, ionized sodium; K^+^, ionized potassium; Ca^2+^, ionized calcium; Cl^−^, ionized chlorine; B, Baseline; I, Induction; R, Recovery; S, Standing; t_30−150_, timepoints in minutes after induction*.

Multiple horses in both groups (MED *n* = 18; XYL *n* = 15) required one or several boluses of ketamine. Two horses in MED and three horses in XYL were administered a single dose of 0.5 or 1.0 mg kg^−1^ thiopental, either to allow intubation (MED *n* = 2; XYL *n* = 2) or because of limb movement during surgery (XYL *n* = 1). There was no significant difference in urine production between the two groups [MED: 6.4 (1.6–14.6); XYL: 5.0 (1.6–15.4) mL kg^−1^ h^−1^; *p* = 0.522].

**Figure 2:**
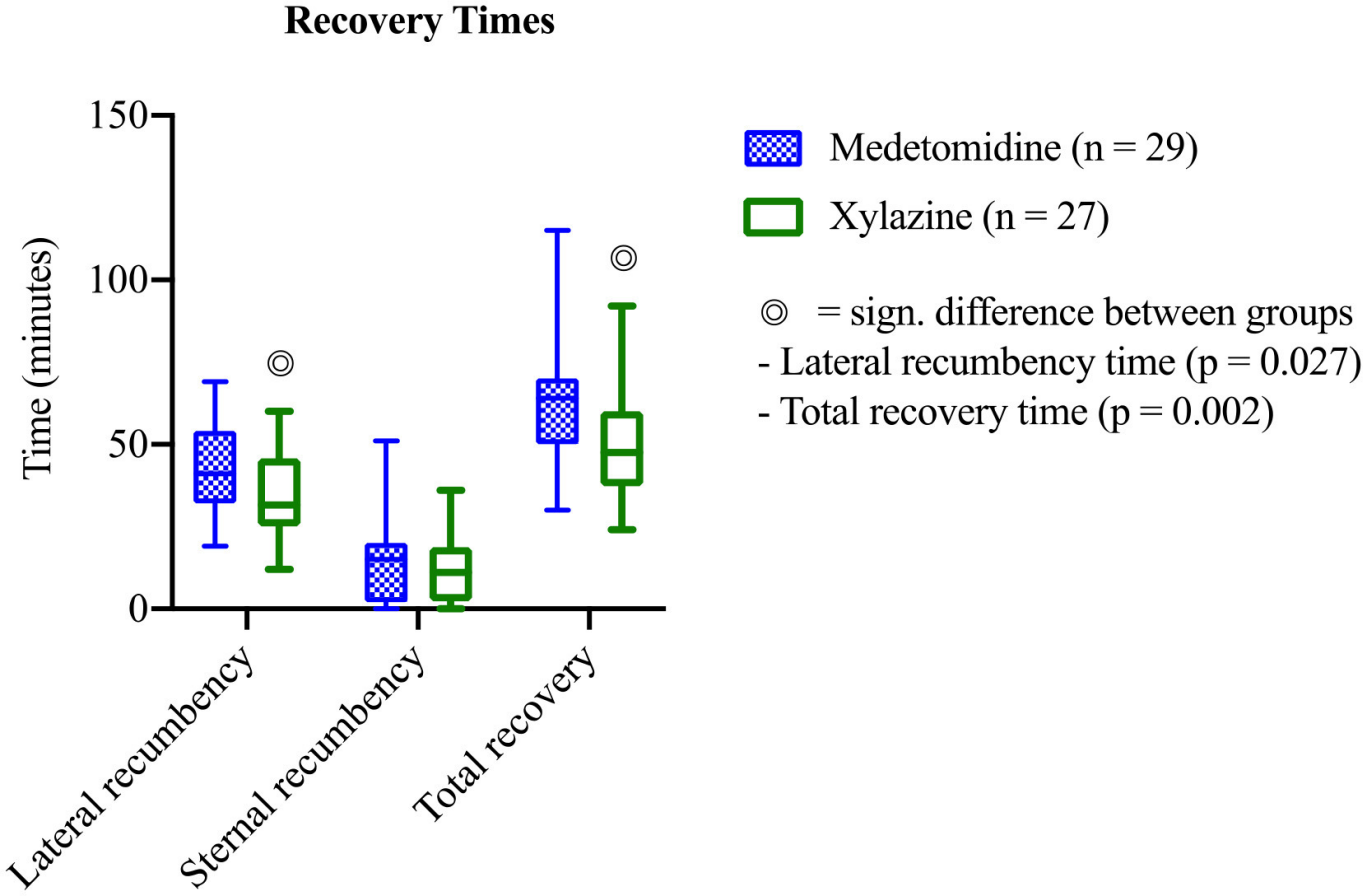
Recovery time in horses after medetomidine isoflurane (group MED) or xylazine isoflurane (group XYL) balanced anesthesia (mean ± SEM). Significant differences between groups is marked with a double circle (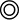).

All horses started to breathe spontaneously within 3–4 min after disconnection from the breathing system and post sedation was administered right thereafter. Total recovery time was significantly longer with medetomidine [MED: 64 (30–115); XYL: 47.5 (24–92) min; *p* = 0.002] (Figure 2). The pivotal phase during recovery was lateral recumbency time, which was significantly longer with medetomidine [MED: 41 (19–69); XYL 31.5 (12–60) min; *p* = 0.027]. No significant difference between groups in the other recorded time spans were detected (in minutes): time to extubation [MED: 15 (11–40); XYL: 15 (8–30); *p* = 0.114]; time from sternal to standing [MED: 15 (0–51); XYL: 11 (0–36); *p* = 0.615]. Recovery was generally of good quality and showed no significant differences between groups in all three scoring systems. In score Sacks et al. (6) where one is best and five is worst recovery quality, 16 horses of group MED and 14 horses of group XYL were classified score 1. The worst recovery scores recorded were from three horses in group MED with a score of 3 and one horse in group XYL with a score of 4 [MED: 1.6 (1–3); XYL: 1.7 (1–4); *p* = 0.837]. In the scoring system by Valverde et al. (31) where recovery quality can range between 8 and 26 plus number of attempts to stand scores were MED: 14 (9–25); XYL: 12 (9–27); *p* = 0.627. Scores of the third scoring system (30) used can range from 8 to 70 plus number of attempts to sternal plus number of attempts to stand [MED: 23 (12–56); XYL: 24 (10–52); *p* = 0.712]. Two horses, one of each group, were administered an additional bolus of either medetomidine or xylazine for recovery, as they showed rapid nystagmus upon arrival in the recovery box which did not cease following the administration of the normal sedative dose.

**Figure 3:**
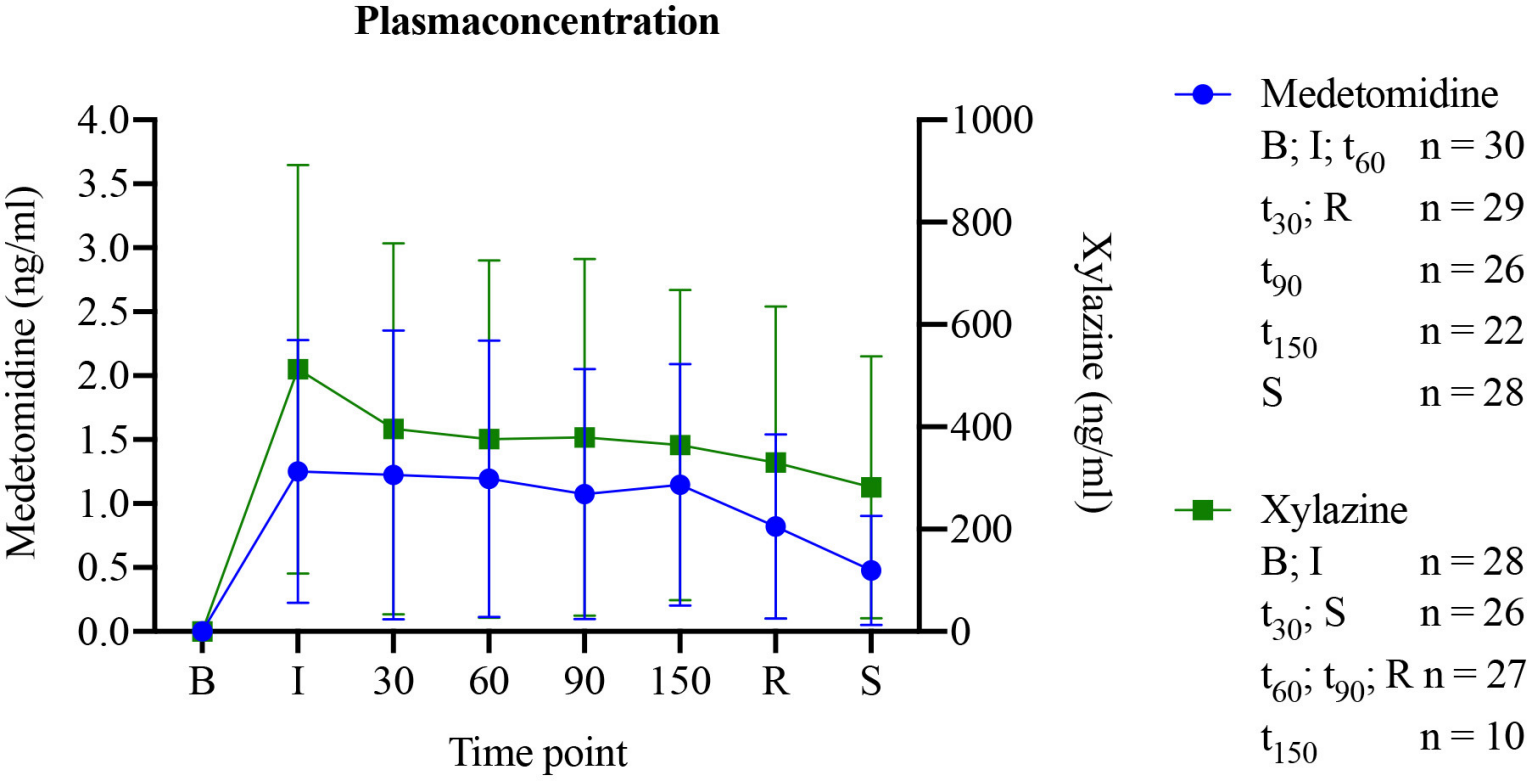
Medetomidine or xylazine plasma concentrations in horses before, during and following medetomidine isoflurane (group MED) or xylazine isoflurane (group XYL) balanced anesthesia (mean ± SEM).

Plasma concentrations of both drugs showed a constant level during general anesthesia (Figure 3).

## Discussion

The current study investigated in horses anesthetized with isoflurane whether the use of medetomidine compared to xylazine for balanced anesthesia resulted in clinically relevant differences in cardiopulmonary function or recovery from anesthesia. The only detectable clinically relevant difference was a shorter recovery following the use of xylazine. Further at some timepoints slightly lower mean arterial blood pressures in combination with a lower need for dobutamine with xylazine were observed. As arterial blood pressures were easily maintained within clinically normal values, the relevance of this finding yet has to be determined.

There is no doubt that even with modern anesthesia techniques and optimized monitoring systems fatality rate in horses is too high and studies to establish safer anesthesia techniques are warranted. Since most anesthetists have stopped to use halothane and use isoflurane or sevoflurane instead (32), less horses have been reported to die intraoperatively, but fatal problems during recovery are predominant (2, 33). Thus, anesthesia techniques resulting in calm recovery should be established without delay.

Alpha_2_-adrenoceptor agonists based PIVA techniques have been used since about two decades (34, 35) and in particular medetomidine and more recently dexmedetomidine have been tested in many horse studies (6, 10, 12). As those drugs are short acting (19, 36, 37) they can be easily titrated to effect and recovery, even after prolonged anesthesia, is usually prompt and of better quality than following lidocaine or ketamine based PIVA protocols (12, 38). As in European countries neither medetomidine nor dexmedetomidine are licensed for food producing animals PIVA protocols including licensed alpha_2_-adrenoceptor agonists are warranted. In conscious horses xylazine, in comparison to the other two for horses licensed alpha_2_-adrenoceptor agonists detomidine and romifidine, has a similar short duration of action as medetomidine and dexmedetomidine (20, 39, 40). Also, distribution half-lives of xylazine are comparable to medetomidine and dexmedetomidine (15, 41). Romifidine and detomidine are longer acting and show more cardiovascular effects than xylazine (20, 42).

We chose to compare medetomidine with xylazine as dexmedetomidine for sedation of clinical patients proven to be not as reliable as medetomidine (6, 43) and dose rates necessary to achieve good sedation before anesthesia induction were considered too variable to be a safe alternative for daily practice.

The present study detected some differences in cardiopulmonary function between the two regimes tested. If the higher arterial blood pressures with xylazine are a result of more vasoconstriction or a better cardiac output will have to be tested in a study with detailed monitoring of cardiopulmonary function. In conscious horses with xylazine at the same dose as used in the present study, effects on cardiopulmonary function were remarkably low (42). It is however noteworthy that with a very low dose dobutamine MAP stayed within targeted values in both groups, but in the XYL group about 30% less dobutamine was needed. We did not use LIDCO to measure cardiac output even though it would be the only safe method to measure cardiac output in clinical cases, as several of the drugs used in the present study have experimentally been shown to have an influence on the measurements of LIDCO (44).

In conscious horses and ponies xylazine and medetomidine at the dose rates used in the current study, cause significant cardiopulmonary depression following bolus administration (19, 20, 42). However, at steady state plasma levels cardiopulmonary function returns to nearly pre-sedation values (19, 42). The plasma levels of both drugs were stable during drug infusion and remarkably similar to the plasma levels measured in horses sedated with similar dose rates of xylazine (26) and slightly higher than what has been reported in standing ponies (19). Although in both groups at the end of CRI following transport into recovery a relevant bolus dose was given to keep the horses sedated and prevent them from an untimely recovery, plasma levels showed a rapid decline, probably as a result of a fast decrease of isoflurane coupled to better liver and general perfusion influencing volume of drug distribution and metabolism.

Acid base balance, electrolytes, lactate and glucose concentrations as well as arterial blood gases remained within clinically acceptable ranges and differences don't seem to be clinically relevant. The amount of urine produced in both groups was considerable and needs to be taken into consideration, when fluid therapy is planned. The use of an intraurethral catheter is advisable in any case.

Bryant et al. (39) showed that medetomidine produces more ataxia than xylazine, so a more ataxic recovery might be expected following medetomidine CRI. Bryant et al. (39) tested medetomidine at a considerably higher dose rate (MED 10 μg kg^−1^ IV) than what was used here for recovery. Therefore, recovery quality in the present study was not different between the groups, as sedative doses used were adequate and not causing additional ataxia. We used 3 different previously described scoring systems (6, 30, 31) to judge recovery as each system evaluates recovery in a slightly different way. We wanted to ascertain that our results are not a consequence of the scoring system used but rather of the medication tested. The groups were similar concerning factors that might affect recovery quality such as anesthesia duration, weight and recumbency during surgery (45, 46). As the incidence of poor recoveries in this study was low and no ASA III-IV horses were included we cannot conclude with only 56 observed recoveries, if the use of either drug is really equal concerning recovery quality. A power analyses of our data revealed that a total of 498 comparable horse anesthetics would have to be investigated to detect with a power of 80% and an alpha error of 0.05, if the incidence of two bad quality recoveries [score 4 and 5 in comparison to 1, 2, or 3 according to Sacks et al. (6)] can be reduced to one, with one or the other PIVA regime.

With xylazine in our study horses remained for a shorter time period in lateral recumbency and recovered overall faster. We can only speculate if this is a result of a quicker redistribution or metabolism of xylazine or a stronger sedative/analgesic effect of the more specific alpha_2_-adrenoceptor agonists medetomidine. Theoretically, recoveries are of better quality if horses don't get up too quickly, as it allows for the inhalant anesthetic to be blown off before the horse attempts to stand up. But with the shorter acting dexmedetomidine recovery from balanced anesthesia was of better quality than following medetomidine PIVA (6), despite otherwise more or less identical effects of the two drugs (at equipotent doses). On the other hand, will prolonged recumbency cause atelectasis and poor perfusion of dependent muscle groups resulting in myopathy and neuropathy. Therefore, the use of xylazine, in particular for prolonged anesthesia's, in compromised patients might prove to be advantageous.

The major limitation of this study is that no sample size calculation with a main outcome was performed. A further weakness is that the depth of anesthesia was judged by a subjective method, but the anesthetist was the same for all procedures and was blinded to treatment. Also, for different procedures horses received two different non-steroidal anti-inflammatories, flunixine and phenylbutazone, but this was equally distributed to the groups.

## Conclusion

In conclusion PIVA with isoflurane and a CRI of xylazine (0.69 mg kg^−1^ h^−1^) compared to medetomidine (3.5 μg kg^−1^ h^−1^) resulted in slightly better MAP, lower need for dobutamine and a faster recovery, whilst overall recovery quality was unaffected.

## Data Availability Statement

The original contributions presented in the study are included in the article/supplementary material, further inquiries can be directed to the corresponding author/s.

## Ethics Statement

The animal study was reviewed and approved by ZH Veterinäramt Zürich. Written informed consent was obtained from the owners for the participation of their animals in this study.

## Author Contributions

AW: study design, acquisition, analysis and interpretation of data and the writing of the manuscript. RB-W: supervised the study and contributed to every part of it. AB: analysis of plasma samples. SR: statistical analysis and interpretation of data. AB, FJ, and MB: collection of data and supervision of clinical study progress. FJ: responsible for randomization of the patients. All authors contributed to the critical revision of the manuscript and approved the final manuscript for publication.

## Conflict of Interest

The authors declare that the research was conducted in the absence of any commercial or financial relationships that could be construed as a potential conflict of interest.
